# Narcissistic consumption in athletes: parallel mediation of brand evangelism between narcissistic personality and hedonic consumption attitude

**DOI:** 10.3389/fpsyg.2026.1773459

**Published:** 2026-06-01

**Authors:** Gokcer Aydin, Ozlem Ece Basoglu, Burak Karababa, Okan Unver, Gokhan Yavuz, Gokhan Aydin

**Affiliations:** 1 Department of Sports Management, Institute of Winter Sports and Sports Sciences, Atatürk University, Erzurum, Türkiye; 2 Department of Physical Education and Sports, Faculty of Sports Sciences, Erzurum Technical University, Erzurum, Türkiye; 3 Department of Recreation, Faculty of Sports Sciences, Çanakkale Onsekiz Mart University, Çanakkale, Türkiye; 4 Department of Physical Education and Sports, Institute of Health Sciences, Kırıkkale University, Kırıkkale, Türkiye; 5 Department of Sports Management, Faculty of Sports Sciences, Atatürk University, Erzurum, Türkiye

**Keywords:** brand, brand evangelism, hedonic consumption attitude, narcissistic personality, sport

## Abstract

**Introduction:**

Narcissistic personality traits have increasingly been associated with identity-driven and pleasure-oriented consumption patterns. In sport contexts, where visibility, achievement, and symbolic recognition are highly salient, personality-based motivations may be closely linked to consumer attitudes. Although prior research has examined the relationship between narcissism and hedonic consumption, limited attention has been given to the psychosocial mechanisms that connect these constructs within athlete populations. This study examines whether brand evangelism mediates the association between narcissistic personality and hedonic consumption attitude in sport, while also investigating whether its subdimensions function symmetrically or asymmetrically in this mediation process.

**Methods:**

Data were collected from student-athletes (*N* = 338; 175 women, 51.8%; 163 men, 48.2%). Validated Turkish versions of the Narcissistic Personality Inventory, Brand Evangelism Scale, and Hedonic Consumption Attitude in Sport Scale were administered. Psychometric adequacy was evaluated through exploratory factor analysis (EFA) and first-order confirmatory factor analysis (CFA) (χ^2^/df = 2.03; RMSEA = 0.055; CFI = 0.931). Mediation analyses were conducted using SPSS 27 and PROCESS Model 4 with percentile bootstrap confidence intervals to estimate indirect associations.

**Results:**

Narcissistic personality was positively associated with brand evangelism (*b* = 0.628, *p* < 0.001) and hedonic consumption attitude (total effect *b* = 0.656, *p* < 0.001). When brand evangelism was included in the model, both evangelism (*b* = 0.412, *p* < 0.001) and narcissism (direct *b* = 0.397, *p* < 0.001) remained significant, indicating partial mediation (indirect effect *b* = 0.259; 95% CI [0.154, 0.370]). In the parallel mediation model, both propaganda (*b* = 0.145; 95% CI [0.084, 0.216]) and attachment (*b* = 0.116; 95% CI [0.048, 0.194]) emerged as significant mediators, with a total indirect effect of 0.261 (*R*^2^ = 0.401). Importantly, the indirect effects of propaganda and attachment were relatively comparable in magnitude, suggesting a symmetrical mediation pattern across the two subdimensions.

**Discussion:**

Interpreted within the framework of ERG theory as a conceptual motivational lens, the findings suggest that growth-oriented and relatedness-oriented tendencies associated with narcissism may be linked to hedonic sport consumption through identity-expressive brand processes. The results further indicate that the propaganda and attachment dimensions operate as distinct mediating pathways rather than uniformly contributing mechanisms. By identifying brand evangelism as a psychosocial mechanism connecting narcissistic tendencies with hedonic consumption attitudes in sport, this study extends personality-based consumption research within sport contexts and offers a more nuanced understanding of how identity-driven motivations are associated with consumer behavior.

## 1 Introduction

In contemporary society, sport consumption extends beyond fulfilling individuals' functional needs and is shaped by multidimensional motivations such as entertainment, pleasure, emotional satisfaction, and social status (Alexandris et al., 2016; Paek et al., 2021). Within the context of sport consumption, consumers now prefer not only products that are oriented toward performance or health but also those that offer aesthetic value, experiential satisfaction, and opportunities for social interaction. At this point, the concept of hedonic consumption emerges, reflecting consumers' pursuit of emotional and experiential value in sport products, equipment, and fan-related goods that go beyond functional needs (Aydin et al., 2026; Elgammal et al., 2025).

Hedonic consumption is closely associated with lifestyle and self-presentation, as sports-related purchases not only provide personal pleasure but also reinforce individuals' social status, sense of group belonging, and emotional attachment to a brand (Kunkel et al., 2014). In particular, the strong emotional bonds fans establish with their teams or sports brands strengthen hedonic consumption behaviors and enhance the effectiveness of marketing strategies (Davis et al., 2019; Elgammal et al., 2025). Therefore, hedonic values in sport consumption should be understood as a multidimensional construct influenced by individual enjoyment, and by social and cultural factors.

Another crucial factor influencing sports consumers' behavior is personality traits. Individual differences significantly affect both sport consumption and hedonic consumption processes, differentiating consumers' preferences and purchasing behaviors (Pourazad et al., 2024). In this regard, narcissism stands out as a personality characteristic grounded in the desire for attention, admiration, and approval from others. Narcissistic individuals seek to display their self-concept and reinforce their social status through the products and services they consume, a phenomenon that is particularly evident in sports contexts (Karababa et al., 2025; Çini and Ceylan, 2025).

The literature emphasizes that narcissistic individuals tend to prefer products that symbolize social status, participate in events that highlight brands, and display more conspicuous consumption behaviors (Mundel et al., 2018; Wetzel et al., 2014). This can be explained through the concept of self-presentation, in which individuals use branded products they own or consume to express themselves and shape how others perceive them. Specifically, items such as fan jerseys or limited-edition sports equipment serve as both sources of personal gratification and instruments of social prestige for narcissistic individuals (Parganas et al., 2015; Margariti et al., 2019). Hence, it can be argued that hedonic consumption is closely related not only to personal satisfaction but also to self-presentation and the pursuit of social approval.

Prior research has linked narcissistic tendencies to conspicuous and status-oriented consumption, showing that narcissistic strategies can predict the display-oriented acquisition of prestige goods (Berry, 2024; Niesiobedzka and Konaszewski, 2022). Separately, marketing scholarship has developed rich discussions of brand-based advocacy behaviors, including brand advocacy and brand evangelism, focusing on why some consumers actively promote and defend brands (Cavadas and Moreira, 2025; Shimul et al., 2026).

However, these two streams are still only partially connected in sport consumption research: studies examining narcissism-related luxury/status consumption among athletes coexist with emerging work on fan/team evangelism (eFANgelism), yet integrative models that explicitly connect narcissistic motives to evangelistic advocacy in sport contexts remain comparatively scarce (Akoglu and Cesur, 2025; Amani, 2023; Cavadas and Moreira, 2025).

Much of the existing literature conceptualizes narcissistic consumption as a direct manifestation of self-enhancement or status signaling, without sufficiently explaining the psychosocial mechanisms through which personality traits may be associated with hedonic consumption attitudes (Fazli-Salehi et al., 2021; Sedikides and Hart, 2022). Similarly, brand evangelism has predominantly been treated as an outcome of brand satisfaction or community identification, rather than as a potential mediating mechanism linking personality dynamics to consumption experiences (Becerra and Badrinarayanan, 2013). This conceptual separation leaves an important gap in understanding how identity-driven motives may be translated into pleasure-oriented sport consumption through relational brand processes.

More specifically, the gap is not only empirical but also conceptual. In adjacent literatures, narcissism is generally treated as a relatively enduring dispositional orientation linked to self-enhancement and impression management (Grijalva and Zhang, 2016), whereas hedonic consumption is framed as an experiential or affective consumption outcome centered on enjoyment, symbolic value, and gratification (Hu and Min, 2022). Brand evangelism, by contrast, has usually been positioned at the relational-behavioral level, capturing how consumers actively advocate, defend, and promote brands in ways that exceed conventional loyalty (Riorini and Widayati, 2015). When these constructs are discussed without making their analytical levels explicit, the model may appear statistically differentiated but theoretically underbounded. Clarifying these boundaries is therefore necessary to explain not merely whether the variables are associated, but how a personality-based motive may be translated into a consumption-related experience through a brand-centered relational process.

From this perspective, the present study treats narcissistic personality as a dispositional antecedent, brand evangelism as a psychosocial and expressive mechanism, and hedonic consumption attitude as an experiential outcome. This distinction helps reduce potential conceptual overlap while preserving the theoretical continuity of the proposed model. It also allows the relationship among the focal variables to be interpreted as a layered process in which self-relevant motives are externalized through brand-oriented advocacy and, in turn, become associated with pleasure-oriented sport consumption.

Building on this distinction, narcissistic individuals' tendency to engage in hedonic consumption for self-presentation is also strongly connected to the concept of brand evangelism (Nandy et al., 2023). Brand evangelism refers not only to consumers' loyalty to a brand but also to their active defense of it, sharing of positive experiences, and recommendation of the brand to others (Cavadas and Moreira, 2025; Çelik and Eş, 2021). In this context, narcissistic consumers use the brands they own or consume as tools to display their social status and strengthen their self-presentation (Siamagka, 2023).

In the sports domain, brand evangelism plays a particularly significant role among fans. Fans not only consume their clubs or sports brands but also voluntarily promote them through social media and other communication channels, enhancing brand visibility while reinforcing their own status within the community (Bhandari et al., 2024; Erdogan et al., 2021; Göktaş, 2023). This process may be particularly relevant for individuals with narcissistic tendencies, as advocacy behaviors provide both social visibility and emotional reinforcement (Sedikides and Hart, 2022). However, although prior research highlights the prominence of evangelistic behaviors in sport (Amani, 2023; Dwyer et al., 2015; Pepur et al., 2023), relatively limited attention has been given to their role as a psychological mechanism linking personality traits to hedonic consumption orientations.

Building on this conceptual gap, the present study examines whether brand evangelism mediates the association between narcissistic personality and hedonic consumption attitude in sport, while also investigating whether its subdimensions—propaganda and attachment—operate symmetrically or asymmetrically within this relationship. By positioning brand evangelism as a psychosocial mechanism rather than merely an outcome variable, the study seeks to extend personality-based sport consumption research beyond direct trait-behavior associations and to offer a more process-oriented explanation of hedonic consumption in sport contexts.

From a broader theoretical standpoint, this research contributes to sport marketing literature by integrating personality dynamics with brand advocacy processes and by clarifying how identity-driven motives may be associated with pleasure-oriented consumption through distinct relational pathways. Moreover, by focusing on athlete-students within a sport-specific context, the study situates personality-based consumption research within a domain where visibility, identity performance, and social recognition are structurally embedded. In this way, the study aims to make an integrated contribution to both the academic literature and sport marketing practice.

## 2 Theoretical framework

### 2.1 ERG (existence-relatedness-growth) theory

Alderfer's ERG theory emerged as a flexible reinterpretation of Maslow's hierarchy of needs. Rather than assuming that individuals move stepwise from one satisfied need to another, ERG proposes that multiple need categories may operate simultaneously and that frustration in one domain can redirect motivational energy toward another (Alderfer, 1972). This dynamic structure makes the theory particularly suitable for understanding complex behavioral domains in which identity, recognition, and experiential gratification intersect (Deci and Ryan, 2000).

ERG groups human motivation into three broad categories: existence, Relatedness, and Growth (Yang and Ling, 2023). Existence needs refer to material security and basic satisfaction (Yu and Hsu, 2022). Relatedness needs involve social connection, recognition, and a sense of belonging (Arogundade and Akpa, 2023). Growth needs concern self-expansion, competence, and the pursuit of personal significance (Yang and Ling, 2023). Unlike rigid hierarchical models, ERG acknowledges that these needs can co-occur, overlap, and interact depending on situational and psychological conditions (Zhuo et al., 2025).

Although ERG theory has most often been used in organizational and work motivation research, its flexible structure makes it suitable for other domains as well (Yang et al., 2011). In particular, it can be extended to consumption contexts where symbolic meaning and social visibility play an important role (Osmanova et al., 2023). In sport consumption settings, products and brands are not only functional objects but also symbols of identity and sources of emotional attachment (Rincón et al., 2023). For this reason, a multidimensional motivational framework such as ERG provides a useful lens for understanding these behaviors.

### 2.2 ERG theory as a conceptual framework

In the present study, ERG theory is not operationalized as a measured construct, nor is it empirically tested as a competing explanatory model. The mediation model estimated in this research includes narcissistic personality traits, brand evangelism (propaganda and attachment), and hedonic consumption attitudes as the focal constructs. ERG is employed solely as a motivational interpretative framework that helps organize and contextualize the observed associations.

More specifically, narcissistic tendencies can be viewed, from an ERG perspective, as reflecting strong growth-oriented motives related to self-expansion, distinction, and visibility (Orth et al., 2024). At the same time, these tendencies may be connected to relatedness needs, particularly when social approval, recognition, and group-based validation are sought through brand affiliation (Nikhashemi et al., 2025; Zeigler-Hill and Dehaghi, 2023). Existence needs are less central in this framework but may still be indirectly implicated when consumption provides immediate affective reassurance or a sense of psychological security through possession and symbolic ownership (Sharifonnasabi et al., 2025).

Within this interpretative structure, brand evangelism may be understood as a psychosocial channel through which identity-expressive brand meanings connect narcissistic motives to hedonic sport consumption (Becerra and Badrinarayanan, 2013). Propaganda behaviors reflect outward-oriented visibility and expressive advocacy, while attachment captures the internalized and identity-based bond with the brand (Rehman et al., 2022). Together, these dimensions help illustrate how motivational orientations may be linked to consumption experiences in sport contexts.

It is important to emphasize that ERG is not positioned as superior to alternative theoretical perspectives such as self-enhancement theory, self-determination theory, symbolic interactionism, or identity signaling frameworks. Each of these perspectives offers valuable explanatory insights. ERG was selected in this study because it provides a parsimonious and integrative motivational taxonomy that allows the findings to be interpreted across broad need domains without overstating causal claims. Accordingly, the empirical conclusions of the study remain grounded in the tested associations, while ERG serves as a conceptual lens through which these relationships are meaningfully organized.

In the present study, ERG theory is not treated merely as a framework for categorizing human needs, but rather as an approach that provides a more suitable explanation for the transformation process examined here. Perspectives such as self-enhancement and symbolic consumption are highly useful for understanding why individuals seek to differentiate themselves, display status markers, or attribute self-related meanings to brands (Belk, 1988; Sedikides and Gregg, 2008). However, these approaches tend to focus primarily either on individuals' internal motivations or on their outward symbolic expressions. In contrast, ERG theory offers a more balanced perspective by allowing the need for personal growth and the need for social connection to be considered simultaneously (Alderfer, 1972). This is particularly important in the context of narcissistic personality. The desire to feel unique or exceptional is rarely independent of the desire to be noticed, validated, and socially recognized by others (Grapsas et al., 2020).

This advantage strengthens the theoretical foundation of the present model. From an ERG perspective, narcissistic tendencies can be understood as reflecting growth-oriented motives related to personal distinction, expansion, and the pursuit of significance. At the same time, these motives are not purely intrapsychic; they are inherently connected to relatedness needs, as individuals seek to be noticed, validated, and socially recognized (Back et al., 2013).

Within this dual-motivational structure, brand evangelism represents the mechanism through which these coexisting needs are externalized in a social context. Specifically, it translates growth-driven desires for distinction into visible advocacy behaviors, while simultaneously satisfying relatedness needs through social recognition and group-based validation.

Hedonic consumption, in turn, reflects the experiential outcome of this process, where these intertwined motives become associated with pleasure, symbolic reward, and emotionally meaningful consumption experiences. In this respect, ERG offers a distinct theoretical advantage by explicitly accounting for the simultaneous activation of growth and relatedness needs, rather than treating them as isolated or sequential drivers of behavior.

Accordingly, ERG does not merely explain that narcissism is associated with consumption, but clarifies how this relationship unfolds through a socially embedded brand process. More specifically, it provides a coherent explanation for how dispositional motives are transformed into consumption attitudes via relational and expressive brand-based mechanisms.

## 3 Development of hypotheses

This study proposes four main hypotheses and two sub-hypotheses, grounded in the theoretical framework and the identified research gap. These hypotheses explain the relationships and mediating effects between narcissistic personality, brand evangelism (with its two sub-dimensions of propaganda and attachment) and hedonic consumption attitude. Each hypothesis is rooted in relevant theory and supported by previous empirical research.

### 3.1 Narcissistic personality and hedonic consumption

Individuals with narcissistic traits are expected to display a higher tendency toward hedonic consumption to enhance their self-image and fulfill their pursuit of pleasure compared to non-narcissistic individuals. This tendency is thought to be related to both Growth and Relatedness needs, as the pursuit of self-actualization and social approval can influence consumption behaviors. The relationship between narcissism and consumption behavior has emerged as a significant topic in psychology and consumer research. The literature indicates that narcissistic personality traits are strongly associated with consumption patterns driven by pleasure and self-enhancement (Kajonius et al., 2015). In this context, narcissistic individuals are shown to be more inclined toward conspicuous consumption and consumption behaviors aimed at positive self-presentation in social environments (Di Pierro and Fanti, 2021).

Moreover, it has been emphasized that narcissism can affect not only symbolic and luxury consumption but also everyday consumption preferences. For example, research suggests that narcissistic individuals' perceptions of food choices may be distorted, thereby increasing hedonic selections (Arpinar, 2022; Yüksel, 2025). Therefore, it can be argued that narcissism may lead individuals toward more pleasure-oriented consumption tendencies.

From an ERG perspective, such pleasure-oriented consumption tendencies may reflect the activation of Growth needs (self-expansion, personal significance) alongside Relatedness needs (social recognition and approval), as narcissistic individuals seek to affirm both their distinctiveness and their social visibility through consumption experiences (Orth et al., 2024).

*H1: Narcissistic personality is positively associated with hedonic consumption attitude*.

### 3.2 Narcissistic personality and brand evangelism

Individuals with narcissistic personality traits are expected to have a higher tendency to defend brands and recommend them to others compared to non-narcissistic individuals. This behavior can be associated with fulfilling the individual's Growth need, as it supports self-actualization and the pursuit of social prestige.

In contemporary contexts, consumption has evolved beyond merely satisfying needs; it has become a domain through which individuals express their identities, pursue pleasure, and demonstrate social status. Within this framework, personality traits -particularly narcissism-play a crucial role in understanding consumer behavior. Narcissism is characterized by an individual's perception of being special, desire for attention, and aspiration to influence others; these tendencies are directly reflected in consumption practices (Cisek et al., 2014).

Narcissistic individuals tend to experience brands not only for their functional benefits but also as extensions of their self-concept. This orientation drives them to advocate for and communicate about their favored brands to others. Empirical evidence shows that narcissistic personality traits strongly shape consumer behaviors, particularly in relation to prestigious brands (Cao and Xu, 2022; Nandy et al., 2023). Therefore, it is expected that a narcissistic personality positively influences brand evangelism.

Within the ERG framework, this association may be interpreted as the outward expression of Growth-oriented motives (prestige, influence, self-enhancement) combined with Relatedness needs, as advocating for a brand enables individuals to gain social acknowledgment and reinforce their perceived significance (Arogundade and Akpa, 2023; Chu et al., 2019; Irawan and Cheng, 2025).

*H2: Narcissistic personality is positively associated with brand evangelism*.

### 3.3 Brand evangelism and hedonic consumption

Consumers' brand loyalty and advocacy behaviors may enhance their likelihood of strengthening social relationships and their sense of belonging. In this regard, individuals who exhibit brand evangelism are expected to display pleasure- and enjoyment-oriented hedonic consumption behaviors more frequently than others. This process can be associated with fulfilling the individual's Relatedness need.

Brand evangelism refers to consumers' extraordinary commitment and advocacy behaviors toward a brand. This attitude often develops alongside hedonic shopping motivations. Previous research has shown that perceptions of brand happiness and prestige increase brand evangelism, which in turn is reflected in pleasure-oriented consumption behaviors (Elgammal et al., 2025; Mansoor and Paul, 2022). Furthermore, hedonic shopping motivations have been found to mediate the relationship between personal values and impulsive purchasing (Demirag and Çavuşoglu, 2020). These findings support the notion that evangelism can strengthen consumers' hedonic consumption attitude.

In ERG terms, brand evangelism may operate as a behavioral channel through which Relatedness needs such as belonging and social connection are translated into emotionally gratifying consumption experiences (Cavadas and Moreira, 2025; Zhu and Park, 2022).

*H3: Brand evangelism is positively associated with hedonic consumption attitude*.

### 3.4 The mediating role of brand evangelism

H4 represents a comprehensive reflection of H1, H2 and H3 hypotheses. In this context, the brand evangelism variable plays a mediating role in the relationship between narcissistic personality and hedonic consumption. Findings in the literature also support this assumption. For example, studies have shown that there is a strong interaction between brand evangelism and hedonic consumption; perceptions of brand happiness and prestige enhance the effect of hedonic motives in this process, thereby strengthening brand evangelism behaviors (Mansoor and Paul, 2022). Similarly, Casaló et al. (2020) found that social media and influencer-based marketing strengthens consumers' tendency toward brand evangelism through hedonic motivations.

These findings suggest that narcissistic personality can reinforce hedonic consumption through brand evangelism. Narcissistic individuals tend to satisfy their need for social visibility, status, and approval through brands. This tendency results in emotional attachment to the brand and advocacy behavior (brand evangelism); thus, this symbolic bond formed with the brand reinforces hedonic consumption attitudes.

Therefore, brand evangelism can be considered the fundamental psychosocial mechanism explaining the relationship between narcissistic personality and hedonic consumption. From an ERG standpoint, brand evangelism may function as a motivational bridge that connects Growth-driven self-expansion motives with Relatedness-based social affirmation processes, thereby linking narcissistic tendencies to hedonic consumption outcomes (Cavadas and Moreira, 2025). Narcissistic personality traits increase individuals' emotional attachment to the brand and their advocacy behavior, thereby elevating their hedonic satisfaction (Mundel et al., 2018). In this regard, the mediating role of brand evangelism emerges as a critical component in understanding narcissistic individuals' identity construction, search for social approval, and pleasure-oriented consumption behavior.

*H4: Brand evangelism statistically mediates the association between narcissistic personality and hedonic consumption attitude*.

### 3.5 Parallel mediation model and associated sub-hypotheses

Examining brand evangelism not only as a holistic construct but also through its sub-dimensions -propaganda and attachment- allows for a more detailed understanding of which specific aspects account for the mediating effect. Accordingly, within the scope of this study, the following sub-hypotheses were developed to test the parallel mediation effects of these two sub-dimensions.

Specifically, the propaganda dimension of brand evangelism refers to an individual's tendency to recommend, defend, and disseminate positive information about a brand to others (Bhandari et al., 2024). In line with this, narcissistic individuals are inclined to satisfy their needs for social approval and visibility through such expressive behaviors (Nandy et al., 2023). Defending or praising a brand can confer social status and attention, which, in turn, may increase the emotional pleasure associated with the brand. Therefore, the propaganda dimension can be considered a potential mediating mechanism that translates the narcissistic desire for social admiration into behavioral expression, thereby indirectly strengthening hedonic consumption tendencies. This process is conceptually aligned with Growth needs expressed through visible self-promotion and Relatedness needs fulfilled through social acknowledgment (Lu et al., 2024).

*H4a: The propaganda dimension of brand evangelism statistically mediates the association between narcissistic personality and hedonic consumption attitude*.

In contrast, the attachment dimension of brand evangelism stems from an individual's emotional sense of belonging to a brand, the perception of the brand as part of one's self-identity, and the development of a psychological closeness to it (Becerra and Badrinarayanan, 2013). Unlike propaganda, which emphasizes external expression, this dimension is associated with the likelihood of fulfilling narcissistic individuals' needs for self-completion and emotional satisfaction. When narcissistic tendencies are combined with strong emotional attachment toward a brand, the symbolic meaning attributed to consumption may increase, thereby reinforcing hedonic consumption behavior (Rindfleisch et al., 2009). Hence, brand attachment can be regarded as a potential emotional mediating mechanism that explains the relationship between narcissistic personality and hedonic consumption. Within the ERG framework, this attachment dimension may be more closely associated with Relatedness needs (belonging and psychological closeness), while simultaneously supporting Growth-related identity reinforcement through symbolic self-extension (Martín-Serrano, 2026).

*H4b: The attachment dimension of brand evangelism statistically mediates the association between narcissistic personality and hedonic consumption attitude*.

Together, the above hypotheses constitute the conceptual model of the present study. Accordingly, these hypotheses will be tested among active athletes who are also students of sport sciences, to provide new insights into the psychological and behavioral mechanisms underlying hedonic consumption in sport. Theoretically, the findings are expected to contribute to the literature on sport consumer behavior, personality traits, and brand evangelism. At the same time, practically, they offer implications for sport marketing strategies targeting athletes and fans with narcissistic tendencies.

**Figure 1:**
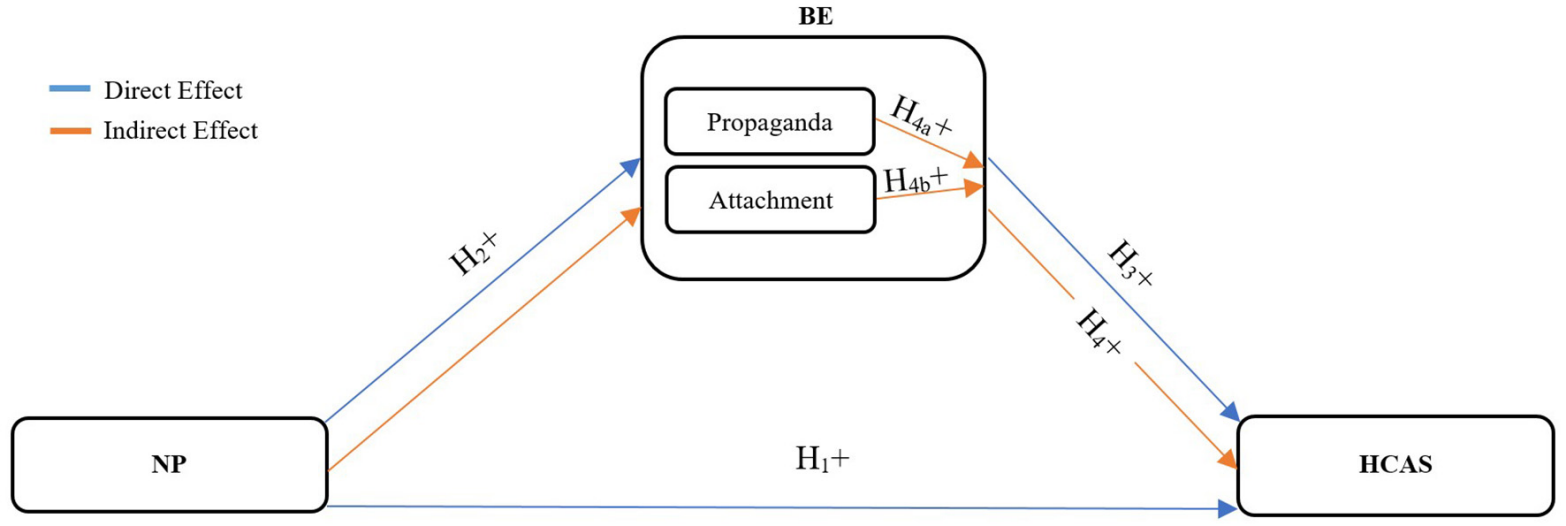
Proposed research model. NP, narcissistic personality; BE, brand evangelism; HCAS, hedonic consumption attitude in sport.

Finally, the proposed research model is illustrated in Figure 1, which visually depicts the study's conceptual framework and the interrelationships among narcissistic personality, brand evangelism (with its two sub-dimensions of propaganda and attachment), and hedonic consumption attitude.

## 4 Method

### 4.1 Research design

This study used a quantitative, cross-sectional survey design. The primary aim was to examine how the study variables co-vary within a specific time window and to evaluate the hypothesized pattern of associations among narcissistic personality, brand evangelism, and hedonic consumption attitudes.

A correlational approach was adopted because it is appropriate for assessing relationships among multiple constructs using regression-based models, while avoiding causal inference from single-wave data (Creswell, 2014; Fraenkel et al., 2012). In line with methodological guidance, the mediation analyses in this study were treated as statistical models of indirect association based on observed covariation, rather than as evidence of temporal or causal ordering (Hayes, 2018).

Accordingly, the results are interpreted as relational and model-based. The cross-sectional nature of the dataset limits conclusions about directionality, and causal claims are avoided throughout the manuscript.

### 4.2 Sample size calculation

In the study, a prior power analysis was conducted using the G^*^Power 3.1 program (Faul et al., 2009) to determine the appropriate sample size. The analysis in this study was conducted within the scope of the PROCESS Model 4, which examines parallel mediation relationships. In the model, which includes three main variables (*X*, *M*_1_, *M*_2_), a small effect size (*f*^2^ = 0.03), significance level (α = 0.05), and 90% statistical power were used as the basis. The analysis was conducted within the “F tests” test family using the “Linear multiple regression: fixed model, *R*^2^ increase” procedure, with three predictors. Based on these parameters, the minimum recommended sample size was estimated as approximately 240 participants, indicating that the sample obtained in this study exceeds the required threshold and ensures sufficient statistical power for investigating the mediation structure.

### 4.3 Participants

In this study, data were collected from student-athletes enrolled at the Faculty of Sports Sciences at Atatürk University who actively participated in licensed athletic activities. Initially, data from 370 students were examined, and after checks for accuracy, consistency, and missing information, a total of 338 student-athletes who met the required criteria were included in the analyses. The sample size in this study exceeds the minimum value required for the specified effect levels and ensures that the analyses achieve sufficient statistical power. The research was approved by the Ethics Committee of the Faculty of Sports Sciences at Atatürk University (decision no: 221, date: 23 October 2025), and informed consent was obtained from all participants prior to data collection.

A purposive (criterion-based) sampling strategy was employed to recruit participants. This approach allows researchers to intentionally select individuals whose characteristics align closely with the aims of the study (Patton, 2015). Given that the research focuses on narcissistic personality traits, brand evangelism, and hedonic consumption attitudes, athletes were considered an appropriate sample group in which these psychosocial dynamics can be observed more clearly. Competitive environments, high performance expectations, public visibility, achievement-oriented motivation, and tendencies toward self-presentation make athletes a population in which narcissistic tendencies may be more pronounced (Roberts et al., 2018). For these reasons, selecting this specific group was deemed more relevant to the study's objectives and more methodologically meaningful than relying on random sampling. Although this sampling strategy restricts generalizability, it provides a sample directly aligned with the study's conceptual focus. Moreover, criterion-based sampling facilitated access to the targeted population and contributed to enhanced data quality (Scielzo et al., 2023).

#### 4.3.1 Inclusion criteria

Participants were included in the study if they met the following conditions:

- Holding a valid and active sports license,
- Enrollment in one of the faculty's undergraduate or graduate programs,
- Completing all measurement instruments fully and accurately,
- Engaging in regular athletic training within the ≥2.
- Providing informed and voluntary consent to participate.

#### 4.3.2 Exclusion criteria

Participants were excluded if they met any of the following conditions:

- Presence of long-term health conditions that prevent consistent sport participation,
- Irregular or discontinuous training or competition schedules,
- Status as former professional athletes not actively competing during their university studies,
- Submission of inconsistent or inaccurate responses that could threaten data quality,
- Failure to complete the informed consent process.

The demographic composition of the student-athletes included in the study is outlined in Table 1 below.

**Table 1:** Demographic characteristics of the participants.

Variable	Category	*n*	%
**Gender**	Female	175	51.8
	Male	163	48.2
**Age**	*18–20*	148	43.8
	*21 and above*	190	56.2
**Type of sport**	Team sports	245	72.5
	Individual sports	93	27.5
**Department**	Coaching education	148	43.8
	Physical education and sports teaching	62	18.3
	Recreation	25	7.4
	Sports management	103	30.5
**Shopping mood**	When happy	305	90.2
	When sad	33	9.8
**Shopping preference**	Online	141	41.7
	In-store	197	58.3
	**TOTAL**	**338**	**100.0**

Percentages are calculated based on the total sample (*N* = 338).

Among the student-athletes, the majority were female (51.8%) and aged 21 and above (56.2%). Most participants engaged in team sports (72.5%) and were primarily enrolled in the Coaching Education program (43.8%). Regarding mood-related purchasing tendencies, the predominant group reported shopping when feeling happy (90.2%). In terms of shopping preferences, the largest proportion of participants preferred in-store shopping (58.3%).

### 4.4 Data collection tools

#### 4.4.1 Personal information form

In the study, the researchers developed a Personal Information Form. The form included questions on variables such as participants' gender, age, field of study, type of sport (team or individual), dominant emotional state during shopping (tendency to shop when happy or sad), and preferred shopping environment (online or physical store).

#### 4.4.2 Narcissistic personality inventory

In this study, the Narcissistic Personality Inventory (NPI), developed by Gentile et al. (2013) and adapted to Turkish culture by (Dogan and Çolak 2020), was used to assess narcissistic personality tendencies among athletes. In the adaptation study, a 13-item, three-factor structure was established, consistent with the original form of the scale: Leadership/Authority, Grandiose/Exhibitionism, and Entitlement/Exploitativeness.

The scale is structured using a five-point Likert-type rating system. Participants were asked to rate the statements on a scale ranging from “1 = Strongly Disagree” to “5 = Strongly Agree”.

In the adaptation study conducted by (Dogan and Çolak 2020), a reliability analysis was performed. The Cronbach's Alpha internal consistency coefficient was found to be 0.84 for the overall scale, and 0.78, 0.72, and 0.61 for the Leadership/Authority, Grandiose/Exhibitionism, and Entitlement/Exploitativeness subscales, respectively.

#### 4.4.3 Brand evangelism scale

The Brand Evangelism Scale developed by Kutlu (2024) was used to determine the levels of brand evangelism among the athletes in the study's sample. The scale consists of 12 items and two sub-dimensions: propaganda and Attachment.

A five-point Likert-type rating scale was used, and participants were asked to rate the statements from “1 = Strongly Disagree” to “5 = Strongly Agree”.

According to the reliability analysis conducted by Kutlu (2024), the Cronbach's alpha coefficient for the Propaganda sub-dimension was determined to be 0.946, for the Attachment sub-dimension 0.911, and for the entire scale 0.899.

#### 4.4.4 Hedonic consumption attitude scale in sports

The Hedonic Consumption Attitude Scale in Sport, developed by Tutar (2023), was used to assess athletes' hedonic consumption attitudes. The scale consists of 18 items across three sub-dimensions: interest, Purchase, and Intrinsic Motivation.

A five-point Likert-type rating scale was used, and participants were asked to rate the statements from “1 = Strongly Disagree” to “5 = Strongly Agree”.

According to the reliability analysis conducted by Tutar (2023), the Cronbach's Alpha internal consistency coefficient for the entire scale was 0.896. Regarding the subscales, Cronbach's Alpha coefficients of 0.892, 0.807, and 0.869 were obtained for the Interest, Purchase, and Internal Motivation subscales, respectively.

### 4.5 Procedure

The data collection process was conducted entirely on a voluntary basis. Participants were informed in advance about the purpose, scope, and implementation of the study. It was clearly emphasized that participation was optional, that the data obtained would be used solely for scientific purposes, and that all personal information would remain confidential.

During the data collection phase, various procedural remedies were applied to minimize the risk of Common Method Variance (CMV). Participants were informed that their responses would remain anonymous, and the order of the scale items was randomized to reduce response patterns influenced by item sequencing. These arrangements were designed to limit systematic response bias and enhance the validity of the findings.

Prior to the administration of the questionnaire, participants were provided with clear instructions on completing the questionnaire form, and standard conditions were ensured for all participants. Researchers prepared a suitable environment to help participants maintain their attention. On average, it took approximately 6 min to complete the data collection tools. Throughout the process, participants were reminded that they were expected to reflect honestly on their experiences, that there were no right or wrong answers, and that what mattered was the sincere expression of personal opinions. These procedures support the assumption that the data obtained reflect participants' genuine perceptions and experiences and that the research was conducted in accordance with ethical principles and scientific standards.

### 4.6 Data analysis

In this study, a quantitative correlational survey design was employed to examine the relationships among the variables (Karasar, 2024).

The analyses were conducted using SPSS 27.0 and Hayes' PROCESS macro (Model 4; Hayes, 2018). The analytical strategy focused on estimating associations among the study variables rather than testing causal effects. The model was specified to examine indirect statistical associations within the hypothesized mediation structure. All indirect paths were estimated using bootstrap confidence intervals, which are recommended for evaluating indirect effects without relying on normality assumptions (Hayes, 2018; Preacher and Hayes, 2008). Accordingly, the mediation results are interpreted as model-based estimates of indirect association rather than evidence of temporal or causal directionality

All scales in the study use a five-point Likert-type rating scale. Since all scales have the same scaling structure and scoring range, it was possible to evaluate the variables at a standard measurement level. Therefore, *z*-score standardization was not required to ensure scale comparability.

#### 4.6.1 Incomplete data analysis

The dataset obtained within the scope of the research was examined for missing observations. According to the Missing Value Analysis (MVA) results in SPSS, no missing data were found in any variable. The missing data rate for all items was 0% (*n* = 370). Upon seeing the warning “There are no missing values” in the SPSS output, Little's MCAR test was not applied. Therefore, no data estimation, correction, or imputation was performed; analyses were conducted on the complete data set.

This indicates that the data's integrity was preserved and that subsequent statistical procedures (normality tests, factor analyses, mediation analyses, etc.) were conducted free of systematic errors arising from missing data.

#### 4.6.2 Extreme value analysis

Outliers in the dataset were examined using z-scores calculated for each variable. The criterion |*z*| > 3.29 (Tabachnick and Fidell, 2013) was applied, and it was determined that no observations fell outside this limit. Accordingly, there are no outliers in the dataset. Multivariate outliers were assessed using the Mahalanobis *D*^2^ statistic. This statistic is a technique proposed to measure the distance between each participant and the variables (Hair et al., 2019). The calculated Mahalanobis *D*^2^ values were compared to the critical value χ^2^(43, *p* = 0.001) = 76.0, where 43 corresponds to the total number of items included in the analysis. It was determined that 32 participants (8.6%) were above this threshold. These observations, while not indicating an error in data entry or systematic bias, were excluded from the analysis to allow for a more robust testing of the multivariate normality assumption and to prevent the covariance structure from being affected by outliers (Kline, 2016; Tabachnick and Fidell, 2013). Thus, the analyses were conducted on 338 valid participants.

In the analyses, it was observed that the extracted observations did not show any particular clustering with respect to demographic variables such as gender, age, or department. This result indicates that no specific group was systematically excluded from the dataset and that the sample is balanced. This type of data cleaning procedure is a standard method recommended to increase the reliability of parameter estimates, particularly in confirmatory factor analysis (CFA) and mediation analyses (Byrne, 2016; Hair et al., 2019).

#### 4.6.3 Normality analysis

The normality assumptions for the three scales used in the study, namely Narcissistic Personality, Brand Evangelism, and Hedonic Consumption in Sport, were examined at both the item and scale total score levels. According to the results obtained at the item level, skewness values ranged from −1.31 to +0.61, while kurtosis values ranged from −1.27 to +1.06. These values fall within the limits suggested in the literature, namely ±2 (George and Mallery, 2019) and ±7 (West et al., 1995), indicating that univariate normality is satisfied.

In the evaluation based on the total scores of the scales, the skewness value for the Narcissistic Personality Scale was −0.056 and the kurtosis value was 0.072; for the Brand Evangelism Scale, the skewness value was −0.140 and the kurtosis value was 0.306; and for the Hedonic Consumption in Sport Scale, the skewness value was −0.567 and the kurtosis value was −0.067. These results indicate that the distributions of all variables fall within statistically normal limits (Tabachnick and Fidell, 2013). These findings show that the data are suitable for parametric analyses and that EFA, CFA, and PROCESS Model 4 mediation analyses can be safely applied. Furthermore, considering that the high sample size (*N* > 200) makes it expected to obtain significant results in the Shapiro–Wilk test (Kline, 2016), both statistical values and visual inspections (histograms, Q–Q plots) were evaluated together, leading to the conclusion that the data showed a distribution close to normality.

#### 4.6.4 Statistical justification for the measurement validation approach

The dimensional structure of the measurement instruments was evaluated through exploratory factor analysis (EFA) followed by confirmatory factor analysis (CFA) using the full dataset (*N* = 338). Although conducting both procedures on the same sample may raise concerns regarding potential overfitting, several methodological considerations support the appropriateness of this approach in the present study.

First, the final CFA model included 40 observed items, resulting in a participant-to-item ratio of approximately 8.45:1. This ratio exceeds commonly recommended thresholds for stable factor estimation, which typically range between 5:1 and 7:1 (Hair et al., 2019; Kline, 2016). In addition, samples exceeding 300 participants are generally regarded as providing stable covariance structures and reliable parameter estimates in structural modeling contexts (Comrey and Lee, 1992; Kline, 2016). Given that the number of estimated parameters remained proportionate to the available observations, the likelihood that model fit indices were artificially inflated due to sample-specific variance patterns is substantially reduced (Brown, 2015).

Second, although the scales employed were previously validated instruments, their application within a specific athlete-student population required renewed examination of their dimensional structure. It is well established that factor compositions may shift when instruments are administered in distinct contextual or cultural settings, particularly when identity-related constructs are involved (Brown, 2015; Hair et al., 2019). In the present study, certain subdimensions demonstrated structural consolidation during EFA. Rather than indicating methodological instability, such consolidation may reflect context-sensitive perception patterns within the athlete population. Therefore, EFA was implemented as a context-sensitive structural assessment rather than a purely confirmatory replication exercise.

Third, the sample represents a relatively specific and homogeneous group of active athlete-students. Dividing the dataset into smaller independent subsamples would have reduced statistical power and potentially increased parameter instability in subsequent CFA procedures. Simulation-based evidence suggests that smaller subsamples may produce less stable loading estimates and greater sampling variability, especially in models involving multiple latent constructs (Wolf et al., 2013). Retaining the full dataset therefore allowed for more stable covariance estimation and more reliable assessment of factor loadings.

Taken together, these statistical and contextual considerations suggest that the use of the full sample for both EFA and CFA constitutes a methodologically defensible strategy within the constraints and characteristics of the present research design.

## 5 Findings

Table 2 presents the results of the Exploratory Factor Analysis (EFA), which was conducted to assess the structural validity of the scales used in the study. All three scales used in the study (NPI, BE, and HCAS) had previously demonstrated acceptable psychometric properties in Turkish samples. In the present study, exploratory factor analyses (EFAs) were first conducted to examine whether the factor structures of these scales replicated in our specific athlete sample and to explore potential cultural reconfigurations of the subdimensions. Subsequently, confirmatory factor analyses (CFAs) were performed on the same sample to evaluate the overall fit of the measurement model and to obtain reliability and validity indices for the subscales that served as observed variables in the mediation models. Before the analyses, the Kaiser–Meyer–Olkin (KMO) sample adequacy test and Bartlett's sphericity test were applied to assess the suitability of the data for factor analysis. The KMO values exceeding 0.80 for all scales, along with the significant Bartlett test result (*p* < 0.001), indicate that the data set is highly suitable for factor analysis (Hair et al., 2019).

**Table 2:** Exploratory factor analysis of NP, BE, and HCAS scales.

Scales	Sub dimension number	Number of items	Factor loading range	Initial eigenvalues	Total variance explained (%)	KMO value	Bartlett's test	*p*
**NP**	LA	9	0.565–0.729	4.980	38.31	0.884	1,836.576	< 0.001
	CESP	4	0.477–0.787	1.216	9.35			
**BE**	Propaganda	6	0.829–0.900	5.384	37.698	0.914	3,714.065	< 0.001
	Attachment	6	0.802–0.897	4.065	36.849			
**HCAS**	IF	7	0.555–0.892	10.162	56.454	0.949	5,174.807	< 0.001
	IPTP	11	0.435–0.818	1.925	10.693			

NP, narcissistic personality; BE, brand evangelism; HCAS, hedonic consumption attitude in sport; LA, leadership and authority; CESP, claim of excessive superiority and privilege; IF, interest and following; IPTP, internal pleasure through purchasing.

### 5.1 Narcissistic personality scale (NP) – exploratory factor analysis

The KMO value for the Narcissistic Personality Scale was 0.884, and the Bartlett test result was χ^2^(78) = 1836.576, *p* < 0.001. The analysis yielded two factors with eigenvalues above 1, which explained 47.67% of the total variance.

The first factor, consisting of nine items, represents an individual's tendency to perceive themselves as a strong, directive, and dominant figure and is therefore termed “Leadership and Authority”. The second factor consists of four items and is called “Claim of Excessive Superiority and Privilege” because it reflects the individual's tendencies to feel special, superior, and entitled.

Although the original scale comprises three dimensions (Leadership/Authority, Grandiosity/ Exhibitionism, and Entitlement/Exploitativeness), this study found that the factors were grouped into two dimensions. This situation can be attributed to the conceptual proximity of some sub-dimensions and cultural factors (Kline, 2016; Tabachnick and Fidell, 2013). The similar perception of statements related to leadership and grandiosity led to the merging of the two dimensions. Therefore, the resulting two-dimensional structure was considered meaningful from both statistical and theoretical perspectives.

### 5.2 Brand evangelism scale (BE) – exploratory factor analysis

The KMO value for Brand Evangelism was 0.914, and the Bartlett test yielded χ^2^(66) = 3,714.065, *p* < 0.001. The analysis yielded two factors with eigenvalues above 1, explaining 74.54% of the total variance.

The first factor consists of six items encompassing behaviors such as recommending, spreading, and defending the brand to others, and is termed “Propaganda”. The second factor consists of six items that express the formation of an emotional bond with the brand and loyalty tendencies and is termed “Attachment”.

The same two sub-dimensions are also present in the original scale, and the factor structure is fully preserved in the current study. The relatively high factor loadings (0.802–0.900) indicate that the scale has strong construct validity (Hair et al., 2019).

### 5.3 Hedonic consumption attitude in sport scale (HCAS) – exploratory factor analysis

In the Hedonic Consumption Scale in Sport, the KMO value was determined to be 0.949, and the Bartlett test result was χ^2^(153) = 5,174.807, *p* < 0.001. The analysis yielded two factors with eigenvalues above 1, which explain 67.15% of the total variance.

The first factor consists of seven items covering interest, attention, and following behavior toward sport and is named “Interest and Following”. The second factor consists of 11 items and includes both purchasing behavior and the internal satisfaction derived from it. Therefore, the second factor is named “Internal Pleasure Through Purchasing”.

In the original scale, there are three sub-dimensions: “Interest,” “Purchase,” and “Internal Motivation.” However, in the current analysis, the second and third dimensions were found to be highly correlated and thus combined into a single factor. This situation may stem from participants perceiving their overall purchasing behavior as driven by both external (product acquisition) and internal (pleasure, enjoyment) motives. This merging indicates a culturally reconfigured factor structure of the scale in the Turkish sample.

KMO values for all scales are at a “very good” level (>0.80), and Bartlett's test is significant, indicating that the data set is suitable for factor analysis. The factor structures obtained demonstrate that the scales measure validly and reliably in the Turkish sample. The merging of some sub-dimensions (e.g., “Purchasing” and “Intrinsic Motivation” in HCAS; “Leadership” and “Grandiosity” in NP) indicates that factors are perceived more holistically in the cultural context.

These results demonstrate that the scales present valid, consistent, and conceptually sound structures within the research sample; they provide a strong foundation for the Confirmatory Factor Analysis (CFA) and mediation analyses to be conducted in the subsequent stage (Byrne, 2016; Hair et al., 2019; Kline, 2016; Tabachnick and Fidell, 2013).

Table 3 presents the results of the confirmatory factor analysis (CFA), which was conducted to test the validity of the measurement model. As the model fit values obtained in the initial study were below the acceptable level, the model was restructured. During this process, items NK1, NK7, and NK10—with standardized factor loadings below 0.60—were removed from the model because they did not adequately represent the underlying latent construct. Low factor loadings indicate that these items conceptually underrepresent the factor, and their removal was deemed appropriate to enhance the reliability and validity of the measurement model (Hair et al., 2019; Kline, 2016).

**Table 3:** Model fit indices of the confirmatory factor analysis.

Model	*X* ^2^	df	χ^2^/df	RMSEA	CFI	GFI	NFI	TLI	IFI
**Model**	1,460.177	719	2.031	0.055	0.931	0.820	0.874	0.926	0.932

RMSEA, root mean square error of approximation; CFI, comparative fit index; GFI, goodness-of-fit index; NFI, normed fit index; TLI, Tucker–Lewis index; IFI, incremental fit index.

Following the removal of items, modification indices (MI) were examined, and specific error covariances deemed theoretically meaningful were added to the model. These covariances were defined only between items belonging to the same sub-dimension and sharing a similar meaning. In this context, covariances were defined between error terms e4 ↔ e5 and e6 ↔ e7 in the Leadership sub-dimension, e21 ↔ e22 in the Propaganda sub-dimension, e23 ↔ e24 in the Attachment sub-dimension, e32 ↔ e33 in the Interest sub-dimension, and e34 ↔ e35 and e37 ↔ e38 in the Purchase sub-dimension. These adjustments were made to explain the common variance arising from the similar wording and measurement contexts of the scale items and do not compromise the theoretical integrity of the model (Byrne, 2016; Hair et al., 2019).

Following the adjustments made, the model's fit values have improved significantly. In the revised model, χ^2^(719) = 1,460.18, *p* < 0.001; χ^2^/df = 2.03; RMSEA = 0.055; CFI = 0.931; TLI = 0.926; IFI = 0.932; GFI = 0.820; NFI = 0.874. A χ^2^/df ratio below three and an RMSEA value below 0.06 indicate that the model fits the data well, while CFI, TLI, and IFI values above 0.90 support the model's overall validity (Hu and Bentler, 1999). GFI and NFI values falling within the range of 0.80–0.90 is a common occurrence in complex models with numerous observed variables and should therefore not be considered a negative impact on the overall fit of the model (Schumacker and Lomax, 2016).

Additionally, the modifications resulted in a decrease in the RMSEA from 0.058 to 0.055 and an increase in the CFI from 0.904 to 0.931. This indicates that the limited and theoretically justified adjustments made enhanced the model's explanatory power and validity. All factor loadings were significant (*p* < 0.001) and generally above 0.60, indicating that the model is adequate with respect to convergent validity.

Lastly, confirmatory factor analysis findings indicate that the measurement model demonstrates a statistically sound level of fit and accurately represents the theoretical structure. Although both exploratory and confirmatory factor analyses were conducted on the same sample due to practical considerations, this approach is widely accepted in scale validation studies when the sample size is sufficiently large. Nevertheless, it is acknowledged that this circumstance may somewhat limit the factor structure's generalizability. Based on the results obtained, the subsequent stage involved conducting reliability (Cronbach's α, CR) and validity (AVE, HTMT, Fornell–Larcker, VIF) analyses of the scales.

Table 4 presents the results of the measurement invariance analysis across gender, conducted using multi-group confirmatory factor analysis. The configural model demonstrated good fit in both groups (χ^2^/df = 1.722, RMSEA = 0.046, CFI = 0.908), indicating that the factor structure was consistent across males and females. Constraining factor loadings to equality did not result in a significant decrease in model fit (ΔCFI = −0.001), supporting metric invariance. Further constraining item intercepts also produced an acceptable model (ΔCFI = −0.003; ΔRMSEA = 0.000), demonstrating scalar invariance.

**Table 4:** Results of measurement invariance analysis (configural, metric, and scalar models) across gender groups.

Model	*X* ^2^	df	χ^2^/df	RMSEA	CFI	NFI	TLI	IFI	ΔCFI	ΔRMSEA
**Configural**	2,461.836	1,430	1.722	0.046	0.908	0.807	0.900	0.909	–	–
**Metric**	2,512.044	1,464	1.716	0.046	0.907	0.803	0.900	0.907	−0.001	0.000
**Scalar**	2,579.531	1,498	1.722	0.046	0.904	0.798	0.900	0.904	−0.003	0.000

RMSEA, root mean square error of approximation; CFI, comparative fit index; GFI, goodness-of-fit index; NFI, normed fit index; TLI, Tucker–Lewis index; IFI, incremental fit index.

Taken together, the results of the configural, metric, and scalar invariance tests provide strong evidence that the measurement model operates equivalently across male and female participants. The factor structure, the strength of item-factor relationships, and the item intercepts are all statistically comparable between groups. Therefore, the measurement instrument demonstrates full invariance across gender, indicating that it measures the underlying constructs in a consistent and unbiased manner for both males and females. This finding confirms that any subsequent comparisons of latent means or structural relationships between the two gender groups are methodologically sound and substantively meaningful.

In this study, confirmatory factor analysis (CFA) was conducted at the first level to examine the mediating role of the sub-dimensions rather than to confirm the entire structure of the scales (Hair et al., 2019; Kline, 2016). This choice enabled each sub-dimension to serve as an independent observed variable in the subsequent parallel mediation analyses conducted within the PROCESS Model 4 framework. This is because the PROCESS method analyzes observed variables (manifest variables) rather than latent variables (Hayes, 2018).

Therefore, evaluating the sub-dimensions as separate constructs both theoretically enabled testing mediation mechanisms at the sub-dimension level and supported a more consistent and interpretable statistical analysis.

The validity and reliability results obtained within the scope of first-level CFA are reported in Table 5. Cronbach's Alpha coefficients for the subscales range from 0.766 to 0.948, and all values are above 0.70 (Nunnally and Bernstein, 1994), indicating that each subscale has sufficient and high internal consistency.

**Table 5:** Reliability and validity results of the measurement subscales.

Variable	α	CR	AVE	1	2	3	4	5	6
1. LA	0.862	0.886	0.494	1					
2. CESP	0.766	0.823	0.556	0.391^***^	1				
3. Propaganda	0.948	0.962	0.783	0.528^***^	0.069	1			
4. Attachment	0.943	0.954	0.729	0.307^***^	0.565^***^	0.140^***^	1		
5. IPTP	0.937	0.932	0.682	0.557^***^	0.355^***^	0.394^***^	0.473^***^	1	
6. IF	0.938	0.931	0.593	0.483^***^	0.127^**^	0.474^***^	0.222^***^	0.698^****^	1

^**^p < 0.01, ^***^p < 0.001; LA, leadership and authority; CESP, claim of excessive superiority and privilege; IF, interest and following; IPTP, internal pleasure through purchasing.

The composite reliability (CR) coefficients ranged from 0.823 to 0.962, with all values above 0.70. The Average Explained Variance (AVE) values ranged from 0.494 to 0.783. These findings indicate that the scales exhibit acceptable convergent validity, in line with the criteria specified by Hair et al. (2019) and Fornell and Larcker (1981). Fornell and Larcker (1981) explicitly note that when AVE is below 0.50 but composite reliability exceeds 0.60, the convergent validity of the construct may still be considered adequate, as the construct explains more variance in its indicators than is attributable to measurement error at the construct level. Although the AVE value of the “Leadership and Authority” sub-dimension (0.494) is slightly below the recommended threshold of 0.50, the high composite reliability coefficient of this sub-dimension (0.886) allows this near-threshold value to be considered appropriate and usable (Malhotra and Dash, 2011).

The AVE value (0.494) for the “Leadership and Authority” sub-dimension fell slightly below the recommended threshold value of 0.50. This outcome appears to be because, despite removing items NK1 and NK7 from the scale during the CFA process due to their low factor loadings, some of the remaining items (e.g., NK9, NK11, and NK13) had factor loadings in the range of 0.60–0.65, which, while acceptable, reduce the average shared variance across indicators. However, methodological literature emphasizes that AVE is a conservative criterion and that marginal deviations from the 0.50 threshold should be interpreted in conjunction with composite reliability and theoretical coherence rather than as an automatic indication of poor convergent validity (Hair et al., 2019; Malhotra and Dash, 2011). In this regard, the slightly lower AVE value for the “Leadership and Authority” construct does not indicate a substantive measurement deficiency but rather reflects the distribution of indicator loadings within an otherwise reliable construct.

When examining the correlation values between the subscales, it is observed that all relationships remain below 0.70. This finding indicates that the constructs exhibit discriminant validity (Fornell and Larcker, 1981). The strongest relationship was found between “Interest and Following” and “Internal Pleasure Through Purchasing,” which are theoretically expected to be linked, with a correlation coefficient of *r* = 0.698 (*p* < 0.001). The moderate level of relationships between the other sub-dimensions supports the notion that each dimension represents a conceptually distinct construct.

In conclusion, the findings from the first-level CFA indicate that the measurement model exhibits high reliability, adequate convergent validity, and good discriminant validity. These findings demonstrate that the mediation analyses to be used in the research are based on a robust measurement foundation.

Table 6 presents the HTMT ratios and the square root of AVE (√AVE) values on the diagonal. In this study, HTMT ratios were computed based on subscale composites given the first-order CFA specification, which is acceptable for assessing discriminant validity at the subdimension level.

**Table 6:** Discriminant validity results based on HTMT ratios and √AVE values.

Constructs	1	2	3	4	5	6
1. LA	**0.703**					
2. CESP	0.477	**0.746**				
3. Propaganda	0.585	0.111	**0.885**			
4. Attachment	0.340	0.666	0.148	**0.854**		
5. IPTP	0.622	0.417	0.419	0.502	**0.826**	
6. IF	0.541	0.186	0.504	0.235	0.744	**0.770**

Bold diagonal values represent the square roots of AVE (√AVE), while off-diagonal values indicate HTMT ratios. LA, leadership and authority; CESP, claim of excessive superiority and privilege; IF, interest and following; IPTP, internal pleasure through purchasing.

According to the criterion proposed by Fornell and Larcker (1981), if the √AVE value of a construct is higher than its correlation coefficients with other constructs, this indicates that discriminant validity is achieved. Upon examination of the table, it can be seen that the √AVE values for all constructs range from 0.703 to 0.885 and that each is higher than the relevant factor's correlation with other constructs. This result confirms that the variables maintain their own conceptual structure and are statistically distinguishable from each other.

All variable pairs in Table 4 had HTMT values below the 0.85 threshold. This finding indicates that the model's discriminant validity is established and that the factors are conceptually distinct (Henseler et al., 2015). The highest correlation was found between the “Interest and Following” and “Internal Pleasure Through Purchasing” variables (HTMT = 0.744). This result reflects the theoretical proximity of these concepts. In contrast, the relatively low correlation between the “Claim of Excessive Superiority and Privilege” and “Propaganda” variables (HTMT = 0.111) supports the notion that these constructs represent distinct conceptual dimensions.

In conclusion, when the Fornell–Larcker criterion and HTMT values are considered together, the model is found to have sufficient discriminant validity. All structures in the measurement model accurately reflect the conceptual domains they represent, and the relationships among them are consistent with theoretical predictions.

Table 7 presents the results of the analysis conducted to assess the potential for common-method bias in the dataset. To assess the potential for common-method bias in self-report data, Harman's single-factor test was conducted using unrotated principal axis factoring (Podsakoff et al., 2003). The analysis yielded six factors with eigenvalues greater than one; the first of these accounted for 35.1% of the total variance. As this value is below the 50% critical threshold, the findings suggest that common method variance is unlikely to represent a dominant single-factor influence in the dataset.

**Table 7:** Harman's single-factor test results.

Extraction method	Number of factors with eigenvalue >1	Variance explained by first factor (%)	Criterion	Result
Principal axis factoring	6	35.1	< 50	No common method bias

Extraction was conducted using unrotated principal axis factoring. The first factor accounted for 35.1% of the total variance, which is below the 50% threshold suggested by Podsakoff et al. (2003).

However, given the documented limitations of Harman's single-factor test in detecting more subtle forms of method variance (Podsakoff et al., 2012), these results should not be interpreted as definitive evidence of the complete absence of common method bias. Rather, they indicate that no substantial single-factor threat was observed within the present data structure.

Before conducting the mediation analysis, multicollinearity among independent variables (Narcissistic Personality, Propaganda, and Attachment) was examined using the Variance Inflation Factor (VIF). As shown in Table 8, all VIF values ranged between 1.238 and 1.595, well below the critical value of 5 (Hair et al., 2019), and all tolerance values were above 0.10. These results indicate that the predictors are statistically independent from each other and that no multicollinearity problem exists within the model.

**Table 8:** Collinearity statistics (VIF values).

Predictors variable	Tolerance	VIF	Criterion	Result
Narcissistic personality	0.627	1.595	< 5	No multicollinearity
Propaganda	0.808	1.238	< 5	No multicollinearity
Attachment	0.755	1.325	< 5	No multicollinearity

Dependent variable: hedonic consumption in sport. All VIF values are below the threshold of five, and tolerance values are above 0.10, indicating no multicollinearity problems (Hair et al., 2019).

Table 8 presents the additional VIF results used to assess the potential presence of common-method variance (CMV). As shown in Table 8, all VIF values were below the conservative threshold of 3.3, indicating that CMV is unlikely to contaminate the results (Kock, 2015). This finding confirms the robustness of the regression and mediation analyses performed in subsequent stages.

In addition to these preliminary diagnostic checks, when the second-order latent mediation model was estimated in the SEM framework, the analysis produced an inadmissible solution because one of the error variances was negative, a condition commonly referred to as a Heywood case. Methodological research has shown that such outcomes may emerge in models involving highly correlated latent constructs, limited sample sizes, weak loadings, or overly complex higher-order structures (Jöreskog, 1999; Kline, 2016; Marsh et al., 2004). These problems are generally interpreted as statistical constraints inherent to the model's estimation rather than evidence of theoretical misspecification (Bentler and Chou, 1987; Chen et al., 2001).

Accordingly, the mediation effect was examined using Hayes' PROCESS Model 4, which operates on observed composite scores and is unaffected by the estimation difficulties that may arise in latent variable models. The PROCESS approach is widely recognized as a robust method for testing indirect effects, particularly due to its bootstrap-based estimation procedures that yield stable results even in the presence of multiple mediators or correlated predictors (Hayes, 2018; Preacher and Hayes, 2008; MacKinnon, 2012). Therefore, the mediation mechanism was assessed at the observed-variable level using this approach.

Table 9 presents the regression results on the mediating role of brand evangelism in the effect of narcissistic personality on hedonic consumption attitudes in sports, at the levels of direct, indirect, and total effects.

**Table 9:** Results of regression and mediation analyses among narcissistic personality, brand evangelism, and hedonic consumption attitude in sport.

Independent variables	*b*	SE	*t*	*p*	LLCI	ULCI
Brand evangelism						
Constant	1.330	0.152	8.712	< 0.001	1.030	1.630
Narcissistic personality	0.628	0.044	14.129	< 0.001	0.540	0.715
	0.610	0.372	199.643	1	336	< 0.001
Hedonic consumption attitude in sport						
Constant	0.853	0.190	4.487	< 0.001	0.479	1.227
Brand evangelism	0.412	0.061	6.720	< 0.001	0.291	0.533
Narcissistic personality (direct effect)	0.397	0.063	6.287	< 0.001	0.272	0.521
Model 2	*R*	*R* ^2^	*F*	df1	df2	*p*
	0.627	0.393	108.636	2	335	< 0.001
Hedonic consumption attitude in sport						
Constant	1.401	0.182	7.674	< 0.001	1.042	1.761
Narcissistic personality (total effect)	0.656	0.053	12.333	< 0.001	0.551	0.760
Model 3	*R*	*R* ^2^	*F*	df1	df2	*p*
	0.558	0.311	152.114	1	336	< 0.001
Indirect Effect			*b*	SE	LLCI	ULCI
			0.259	0.055	0.154	0.370

Model 1 presents that the effect of narcissistic personality on brand evangelism is significant and positive. According to regression analysis findings, narcissistic personality strongly predicts brand evangelism (*b* = 0.628, SE = 0.044, *t* = 14.129, *p* < 0.001). This finding reveals that individuals with high narcissistic tendencies tend to identify more strongly with brands and exhibit more intense brand defense and promotion behaviors. The model was found to be generally significant (*F*(1,336) = 199.643, *p* < 0.001) and explains 37.2% of the variance (*R*^2^ = 0.372). The obtained regression coefficient (*b* = 0.628) indicates a large effect according to Cohen's (1988) classification. This result shows that narcissistic personality strongly predicts brand evangelism behaviors and that brand-related advocacy behaviors have a personality-based structure.

Model 2 presents the combined effects of narcissistic personality and brand evangelism when the attitude toward hedonic consumption in sports is added as a dependent variable. According to the analysis results, both variables have significant, positive effects on hedonic consumption attitude toward sports (brand evangelism: *b* = 0.412, SE = 0.061, *t* = 6.720, *p* < 0.001; narcissistic personality: *b* = 0.397, SE = 0.063, *t* = 6.287, *p* < 0.001). The model is generally significant (*F*(2,335) = 108.636, *p* < 0.001) and explains 39.3% of the variance (*R*^2^ = 0.393).

Model 3 presents the total effect of narcissistic personality on hedonic consumption attitudes in sports. According to the analysis results, this effect was significant and positive (*b* = 0.656, SE = 0.053, *t* = 12.333, *p* < 0.001). The model was found to be significant (*F*(1,336) = 152.114, *p* < 0.001) and explained 31.1% of the variance (*R*^2^ = 0.311). The obtained coefficient *b* = 0.656, represents a large effect size according to Cohen's (1988) classification. This result shows that narcissistic personality is a strong predictor of hedonic consumption attitudes in sports. Still, when brand evangelism is added to the model, this effect partially diminishes, revealing a partial mediating effect.

According to the mediation analysis results, brand evangelism plays a significant partial mediating role in the effect of narcissistic personality on hedonic consumption attitudes in sports. The indirect effect coefficient is *b* = 0.259 (SE = 0.055, LLCI = 0.154, ULCI = 0.370), and the confidence interval does not include zero, indicating that the indirect effect is statistically significant. According to Preacher and Kelley's (2011) classification, the magnitude of this indirect effect (*b* = 0.259) corresponds to a large mediation effect (*b* ≥ 0.25).

Furthermore, the proportion of the indirect effect within the total effect was calculated as PM = 0.259 / 0.656 ≈ 0.395. This proportion indicates that approximately 39.5% of the effect of narcissistic personality on the hedonic consumption attitude in sports occurs through brand evangelism.

The magnitude of the indirect effect was also assessed using the standardized κ^2^ coefficient. The approximate κ^2^ value of 0.39 obtained indicates a large effect size according to Preacher and Kelley's (2011) classification (κ^2^ ≥ 0.25). This result reveals that the indirect effect is not only statistically significant but also a powerful psychological effect with high explanatory power.

When the three models are evaluated together, it is seen that the effect of narcissistic personality on hedonic consumption attitude in sports is significant both directly (*b* = 0.397) and indirectly (*b* = 0.259). While the total effect is strong (*b* = 0.656), the indirect effect is large (κ^2^= 0.39, PM ≈ 0.40).

PROCESS analysis results indicated that the indirect effect was significant based on the bootstrap confidence intervals. This finding shows that brand evangelism performs a partial mediating role in the relationship between narcissistic personality and hedonic consumption attitudes in sport. Considering that the bootstrap method is regarded as one of the most powerful and reliable approaches for evaluating indirect effects (MacKinnon et al., 2004; Preacher and Hayes, 2008), these results demonstrate that the mediation mechanism is supported in a statistically robust manner. Thus, despite the estimation limitation encountered in the second-order latent SEM model, the mediation structure was tested at the observed-variable level within a methodologically appropriate and literature-consistent framework.

Table 10 presents the regression results regarding the parallel mediating roles of the brand evangelism subdimensions (Propaganda and Attachment) in the effect of narcissistic personality on hedonic consumption attitudes in sports, including direct, indirect, and total effects.

**Table 10:** Results of Parallel Mediation Analysis of the Effects of Brand Evangelism Subdimensions (Propaganda and Attachment) on the Relationship Between Narcissistic Personality and Hedonic Consumption Attitude in Sport.

Independent variables	*b*	SE	*t*	*p*	LLCI	ULCI
Propaganda						
Constant	2.329	0.205	11.349	< 0.001	1.926	2.733
Narcissistic personality	0.523	0.059	8.751	< 0.001	0.405	0.640
Model 1	*R*	*R* ^2^	*F*	df1	df2	*p*
	0.430	0.185	76.587	1	336	< 0.001
Attachment						
Constant	0.330	0.245	1.349	0.178	−0.151	0.813
Narcissistic personality	0.733	0.071	10.271	< 0.001	0.593	0.874
Model 2	*R*	*R* ^2^	*F*	df1	df2	*p*
	0.488	0.239	105.504	1	336	< 0.001
Hedonic consumption attitude in sport						
Constant	0.703	0.202	3.481	< 0.001	0.306	1.101
Propaganda	0.277	0.045	6.078	< 0.001	0.187	0.366
Attachment	0.158	0.038	4.143	< 0.001	0.083	0.233
Narcissistic personality (direct effect)	0.395	0.062	6.288	< 0.001	0.271	0.518
Model 3	*R*	*R* ^2^	*F*	df1	df2	*p*
	0.633	0.401	74.620	3	334	< 0.001
Hedonic consumption attitude in sport						
Constant	1.401	0.182	7.674	< 0.001	1.042	1.761
Narcissistic personality (total effect)	0.656	0.053	12.333	< 0.001	0.551	0.760
Model 4	*R*	*R* ^2^	*F*	df1	df2	*p*
	0.558	0.311	152.114	1	336	< 0.001
Indirect effect			*b*	SE	LLCI	ULCI
	Propaganda		0.145	0.033	0.084	0.216
	Attachment		0.116	0.036	0.048	0.194
	TOTAL		0.261	0.055	0.157	0.378

Model 1 presents the effect of narcissistic personality on propaganda, one of the two sub-dimensions of brand evangelism. According to the analysis results, the effect of narcissistic personality on propaganda was found to be positive and significant (*b* = 0.523, SE = 0.059, *t* = 8.751, *p* < 0.001). The model explains 18.5% of the variance (*R*^2^ = 0.185). According to Cohen's (1988) classification, this value represents a medium effect size.

Model 2 presents the effect of narcissistic personality on brand attachment, the second sub-dimension of brand evangelism. According to the analysis findings, the effect of narcissistic personality on brand attachment is positive and significant (*b* = 0.733, SE = 0.071, *t* = 10.271, *p* < 0.001). The model explains 23.9% of the variance (*R*^2^ = 0.239). According to Cohen's (1988) classification, this value represents an effect size between medium and large.

Model 3 presents that the variables of propaganda (*b* = 0.277, *p* < 0.001) and attachment (*b* = 0.158, *p* < 0.001) significantly predict hedonic consumption attitudes in sports. Furthermore, the direct effect of narcissistic personality remained significant (*b* = 0.395, *p* < 0.001). The model's overall explanatory power is 40.1% (*R*^2^ = 0.401). When mediators were added, the effect of narcissistic personality decreased, supporting a partial mediation effect.

Model 4 presents the total effect of narcissistic personality on hedonic consumption attitudes in sports (*b* = 0.656, *p* < 0.001; *R*^2^ = 0.311). According to Cohen's (1988) classification, this value represents a large effect size.

Findings related to indirect effects indicate significant mediation through both propaganda (*b* = 0.145, SE = 0.033, CI [0.084, 0.216]) and attachment (*b* = 0.116, SE = 0.036, CI [0.048, 0.194]). The total indirect effect is *b* = 0.261 (SE = 0.055, CI [0.157, 0.378]), and the proportion of the indirect effect in the total effect is calculated as PM = 0.261 / 0.656 ≈ 0.40. This value indicates that approximately 40% of the effect of narcissistic personality on hedonic consumption in sports occurs indirectly through the two sub-dimensions of brand evangelism.

The magnitude of the indirect effect was calculated as κ^2^ = 0.39 and was evaluated as a large level of mediating effect according to the classification proposed by Preacher and Kelley (2011) (κ^2^ ≥ 0.25 = large). This finding indicates that the two sub-dimensions of brand evangelism, namely propaganda and attachment, are strong mediators with high explanatory power in the relationship between narcissistic personality and hedonic consumption in sports.

In addition, when the findings in Tables 7, 8 are examined, it is observed that in the simple mediation analysis, the indirect effect of narcissistic personality on hedonic consumption attitude in sport through brand evangelism was 0.259. In contrast, in the parallel mediation analysis, in which the two subdimensions of brand evangelism (Propaganda and Attachment) were simultaneously included in the model, the total indirect effect was 0.261.

This very small difference arises from decomposing the overall brand evangelism variable into its subdimensions and from the estimation procedure used in parallel mediation models. Such minor differences between simple and parallel mediation models are methodologically expected. In the simple mediation model, the indirect effect reflects the mediating role of brand evangelism as a single, unified construct. In contrast, in the parallel mediation model, each subdimension (Propaganda and Attachment) is estimated separately, and the total indirect effect is obtained by summing their unique contributions. As noted by Kane and Ashbaugh (2017), decomposing a single mediator into multiple subcomponents within a parallel mediation framework allows the exceptional contribution of each mediator to be captured. Therefore, slight differences between total indirect effect coefficients obtained from parallel mediation models and those from simple mediation analyses are expected and indicate a more detailed representation of the mediation mechanism.

**Figure 2:**
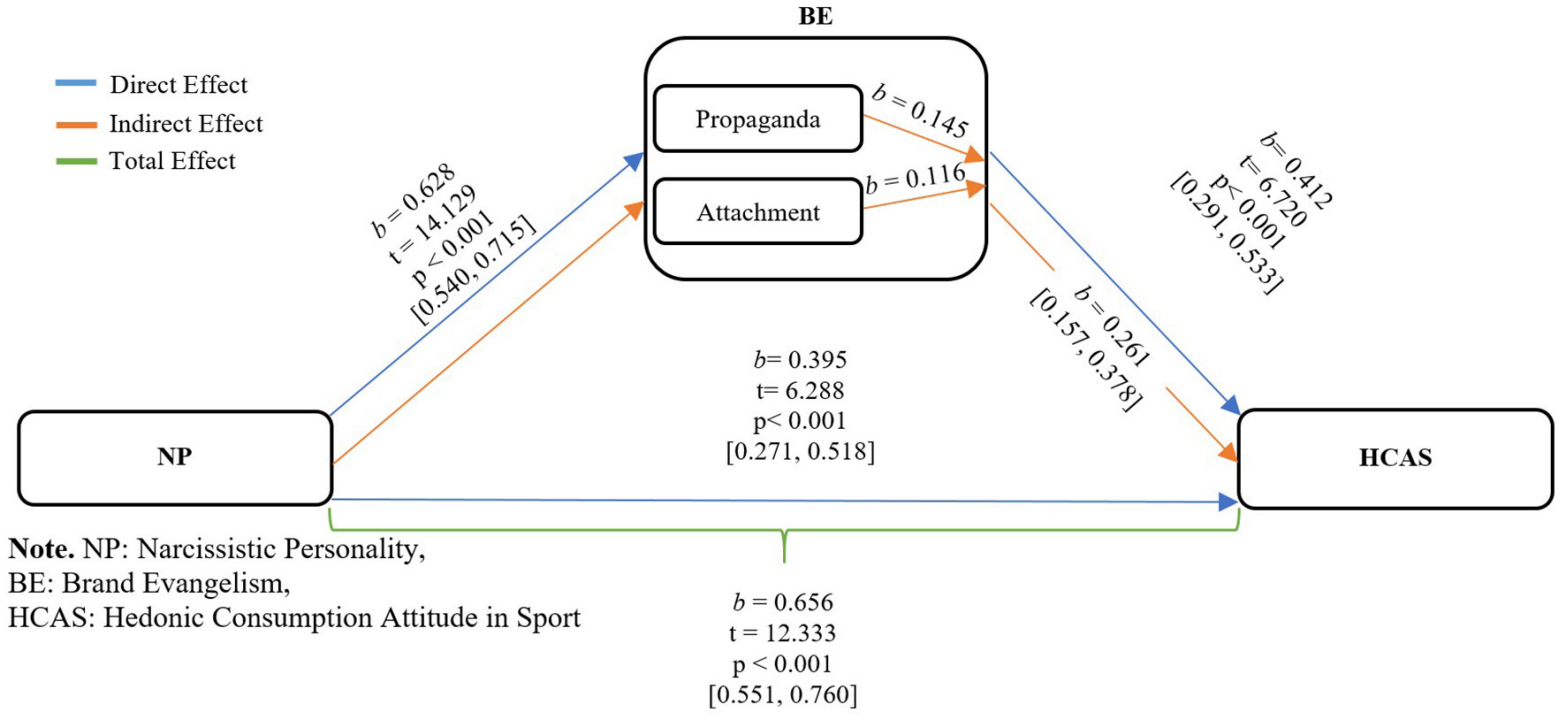
Parallel mediation model of brand evangelism (propaganda and attachment) in the relationship between narcissistic personality and hedonic consumption attitude in sport. NP, narcissistic personality; BE, brand evangelism; HCAS, hedonic consumption attitude in sport.

Figure 2 visually presents the results reported in Tables 9, 10, illustrating the parallel mediation effects of brand evangelism (sub-dimensions: propaganda and attachment) on the relationship between narcissistic personality and hedonic consumption attitude in sports. The figure summarizes both direct and indirect effects, showing that both sub-dimensions exert significant indirect effects, with a total indirect effect of 0.261 (bootstrap 95% CI [0.157, 0.378]). Since the direct effect of narcissism remains significant, the results indicate a partial mediation structure. To maintain visual clarity, significance indicators (*p*-values, confidence intervals) for the sub-dimensions are not displayed in the figure; the corresponding *b* coefficients and LLCI-ULCI values are reported in Table 10, while the summary of the simple mediation model is provided in Table 9.

## 6 Discussion

This study examined the associations between athletes' narcissistic personality traits and hedonic consumption in sports, as well as the mediating role of brand evangelism in this relationship. The findings revealed that narcissistic personality was significantly and strongly associated with both brand evangelism and hedonic consumption in sports. Furthermore, brand evangelism statistically mediated the association between narcissistic personality and hedonic consumption in sports. These results provide valuable insights into how athletes' personality structures may be linked to consumption through the emotional and symbolic relationships they form with brands. In particular, the narcissistic personality's self-aggrandizement, achievement orientation, and search for visibility appear to be associated with identity construction and hedonic gratification through brands. This relationship may be interpreted through self-presentation and self-verification processes (Goffman, 2023; Sedikides and Gregg, 2008), where narcissistic individuals may engage with brands as symbolic resources through which psychological rewards and social approval are experienced.

Narcissistic tendencies in sports may be related to individuals' attitudes, motivations, and consumption behaviors toward sports. Narcissistic athletes often seek success, attention, and social recognition (Manley et al., 2019). While the narcissistic admiration dimension has been associated with performance-related psychological traits such as self-confidence and mental toughness (Nevicka et al., 2023), the narcissistic competitive dimension has been linked to excessive self-centeredness and dependency tendencies (Altunhan and Tazegül, 2023). This two-way structure may be interpreted through the mechanism of self-worth being regulated by external validation (Deci and Ryan, 2000). To the extent that individuals validate their own worth through social approval, they may be more inclined to engage in symbolic means, such as success and brand use. The findings of (Cin and Yaşar 2022) and Hazar et al. (2024) also suggest that narcissistic individuals' pursuit of happiness, exercise addiction, and visibility may reflect this external reward mechanism. Within the scope of the present study, these dynamics may help explain why narcissistic tendencies are associated with stronger engagement in brand-related behaviors and consumption experiences in sport contexts.

Our research indicated a strong statistical association between narcissistic personality and brand evangelism (β = 0.628). This finding is consistent with consumer-person relationship models in the literature (Belk, 1988; Tajfel and Turner, 2004) and the self-presentation-oriented consumption behaviors of narcissistic personality (Sedikides and Gregg, 2008). Brand evangelism involves an individual's intense emotional identification with the brand, characterized by voluntary behaviors such as brand advocacy, praise, and disparagement of competing brands (Becerra and Badrinarayanan, 2013; Dwyer et al., 2015). Narcissistic individuals may use brands as an extension of their social identities, with higher levels of perceived self-worth expressed through external symbols. This may be interpreted in light of their motivations for self-aggrandizement and creating social difference (Sedikides and Strube, 1997; Campbell and Foster, 2011) which may coincide with the symbolic value of the brand. Indeed, Li et al. (2022) showed that self-brand connection increases evangelistic behavior. This finding suggests that the self-glorification tendency in narcissistic personality is associated with stronger emotional identification with the brand and a higher likelihood of evangelistic behavior.

The significant effect of brand evangelism on hedonic consumption in sport (β = 0.412) indicates a meaningful statistical association between emotional brand engagement and hedonic gratification. This finding may be interpreted through the emotional reward, self-satisfaction, and self-reinforcement mechanisms underlying hedonic consumption (Holbrook and Hirschman, 1982). In studies conducted by Holbrook and Hirschman on a general consumer population, it was stated that emotional experiences are more influential than cognitive processes in consumption decisions. Similarly, studies conducted by Kim et al. (2022) and Kim et al. (2017) on university students and sporting goods consumers revealed that hedonic gratification supports psychological well-being and that brand experiences are associated with positive affect. These results are consistent with the findings of the current study, as the emotional bonds athletes form with brands may extend beyond performance or aesthetic expectations, potentially reflecting needs for self-esteem reinforcement and social belonging. Zhigang et al. (2022) emphasized that social ties are associated with stronger belonging-based intrinsic motivation in brand interactions. In this context, brand evangelism may function not only as brand advocacy behavior in athletes but also as a psychological satisfaction mechanism linked to the narcissistic personality's quest for social acceptance and appreciation.

Propaganda and attachment, the sub-dimensions of brand evangelism, were found to statistically mediate the association between narcissistic personality and hedonic consumption. Propaganda may reflect narcissistic individuals' outward self-presentation and desire for admiration, while attachment may represents the internal identity satisfaction associated with identifying with the brand. These different orientations can be interpreted in relation to self-presentation and self-integrity processes (Sedikides and Strube, 1997). Findings from the studies by Becerra and Badrinarayanan (2013) and Khandai et al. (2025) suggest that these psychological processes are internal mechanisms that support evangelism. The emotional evangelism clusters described by Cavadas and Moreira (2025) and Dwyer et al. (2015) also confirm narcissistic individuals' tendency to maintain self-integrity through identification. Thus, narcissistic athletes' use of the brand as a symbol of their own success appears to be associated with their needs for both internal consistency and external validation.

Building on these findings, it is useful to consider more closely how the motivational logic proposed by ERG theory can help interpret the psychological pathway identified in this study. ERG theory conceptualizes human motivation through three interconnected need domains, existence, relatedness, and growth, rather than assuming a strict hierarchical progression (Alderfer, 1972). Within this framework, narcissistic tendencies may be associated primarily with growth-oriented motives, including the pursuit of distinction, recognition, and personal significance (Orth et al., 2024). In highly visible social environments such as sport, where success, prestige, and symbolic performance are socially valued, these motives may encourage individuals to seek resources that reinforce their self-concept. Sport-related brands can serve precisely this role because they operate not only as functional objects but also as symbolic markers of identity, affiliation, and achievement (Belk, 1988; Rincón et al., 2023). From this perspective, narcissistic personality traits may increase athletes' engagement with brands as identity-expressive symbols that support the pursuit of growth-related psychological needs.

At the same time, the mediating role of brand evangelism may reflect the activation of relatedness needs within the ERG framework. Relatedness needs involve social belonging, recognition, and validation within interpersonal contexts (Ryan and Deci, 2024; Yang and Ling, 2023). Evangelistic behaviors, such as recommending, defending, or publicly endorsing a brand, create opportunities for individuals to gain acknowledgment from their reference groups. In sport settings, where identity and group affiliation are highly visible, these actions may strengthen social visibility while reinforcing perceived belonging within sport communities. In this sense, brand evangelism may function as a relational mechanism through which narcissistic growth-oriented motives are translated into socially reinforced consumption experiences. This interpretation aligns with research indicating that brand advocacy and evangelistic behaviors often emerge when consumers integrate brands into their social identities and interpersonal interactions (Becerra and Badrinarayanan, 2013; Cavadas and Moreira, 2025).

Furthermore, distinguishing between the propaganda and attachment dimensions of brand evangelism provides additional insight into the psychological structure of this mediation process. The propaganda dimension appears to reflect outward-oriented identity signaling, where individuals actively promote or defend brands as visible extensions of their self-concept. In contrast, the attachment dimension represents a more internalized psychological bond with the brand and reflects emotional closeness together with symbolic self-extension (Rindfleisch et al., 2009). Considering these two mechanisms together suggests that narcissistic athletes may relate to brands through both external self-presentation and internal identity integration processes. Consequently, hedonic sport consumption may arise not only from the pursuit of pleasure but also from the symbolic satisfaction associated with expressing and reinforcing one's identity through brand relationships.

### 6.1 Theoretical contributions

This study presents a psychosocial framework that integrates narcissistic personality theory with sports consumer behavior, highlighting how emotional and symbolic brand relationships may statistically mediate personality-based consumption patterns. Findings indicate that narcissistic traits are associated with hedonic consumption through brand-based identity construction, which appears to reflect self-presentation rather than purely utilitarian motives. The dual mediating effect of propaganda and attachment variables in this process helps clarify the pathway through which narcissistic tendencies are associated with hedonic consumption, with propaganda representing outward self-aggrandizement and attachment reflecting internal self-definition. This distinction contributes to models of evangelical behavior (Becerra and Badrinarayanan, 2013; Cavadas and Moreira, 2025) and supports the notion that brands function as symbolic extensions of the athlete's self. By demonstrating statistically significant associations between the admiration-competition components of narcissism and hedonic consumption mechanisms, this study expands theoretical understanding in both sport psychology and consumer-brand relationships. Brand evangelism may be conceptualized as a psychological pathway linking personality dynamics and consumption-related satisfaction.

### 6.2 Practical implications

The findings have direct implications for sport marketers, brand strategists, and sport psychologists. Brands that target athlete consumers may benefit from emphasizing symbolic value, exclusivity, and achievement narratives that resonate with narcissistic motives for recognition and admiration. Marketing campaigns that enable public self-expression and social visibility may strengthen athletes' identification with brands, potentially enhancing advocacy and loyalty. Coaches and sports organizations may strategically leverage brand identification to support motivation and commitment, while promoting ethical awareness to avoid materialistic excesses. For sustainable sports branding, authenticity and trust must be prioritized so that admiration-based evangelism is more likely to evolve into positive brand advocacy rather than rivalry or aggression. Understanding brand evangelism as a mediating mechanism may assist practitioners in designing digital brand communities and engagement programs that channel athletes' competitive energy into constructive advocacy and long-term brand attachment.

## 7 Conclusion

In summary, this study found that narcissistic personality traits were significantly associated with hedonic consumption in sports, while brand evangelism partially mediates this relationship through its propaganda and attachment dimensions. Narcissistic athletes, motivated by the desire for visibility, admiration, and achievement, appear more likely to form strong emotional and symbolic bonds with brands. These brand connections may reinforce their self-concept and be associated with hedonic gratification by fulfilling needs for self-expression and social validation.

The results emphasize that hedonic consumption in sports may reflect not only individual preferences or material motivations, but also reflects deeper psychosocial processes. In particular, the mediating role of brand evangelism suggests that emotional and symbolic identification with brands may serve as a psychological mechanism that channels narcissistic drives into consumer behavior. By highlighting this indirect pathway, the study contributes to a more nuanced understanding of how personality traits are related to athletes' brand relationships and consumption patterns within the framework of sports psychology and consumer behavior theories.

From a broader perspective, these findings highlight the increasing importance of brands as identity resources in contemporary sports culture. For athletes, brands are not only performance-related tools but also extensions of self-presentation and social recognition. Understanding this connection provides valuable insights for sport marketers and psychologists seeking to balance athletes' identity needs with ethical, authentic, and sustainable branding practices.

From an ERG perspective, these findings may be interpreted as reflecting the interplay between Growth-oriented motives (self-expansion, achievement, personal significance) and Relatedness needs (social recognition and belonging) in shaping consumption-related behaviors among athletes. Although ERG was not empirically tested as a latent construct within the present model, it provides a structured motivational lens through which the observed associations can be meaningfully organized. In this sense, the mediation pathway identified in the study can be understood as a potential psychosocial process linking need-based motivational tendencies to identity-expressive consumption in sport contexts.

## 8 Limitations and future research

This study has several limitations, and the findings should be evaluated within this framework. First, the study employed a cross-sectional design. This limits inferences about causal relationships between variables. Accordingly, the findings should be interpreted as relational rather than directional. Future studies using longitudinal or experimental designs will contribute to testing the causal direction between variables and their consistency over time.

Second, the study sample consisted of active student-athletes studying at a single university. This limits the generalizability of the results to individuals of different ages, regions, or competitive levels. Including participants from different universities and performance levels in future studies would strengthen external validity.

Third, the distribution of participants by sport and gender was not fully balanced. This imbalance could affect the robustness of the mediation model. Although these proportions are presented in detail in the methodology section, the number of participants was not equalized by sport and gender during the data collection phase. The primary reason for this is that, during the voluntary data collection process, access to participants from certain branches and genders was restricted. It is recommended that future research utilize sample planning to mitigate such imbalances.

Fourth, the study was conducted solely on a Turkish sample. Because cultural context can influence the perception and expression of variables such as narcissism or brand evangelism, comparative studies across different cultures are crucial for testing the cross-cultural validity of the findings.

Fifth, the study only considered brand evangelism as a mediator variable. Future studies could further explore the inclusion of other psychological variables, such as self-esteem, materialism, social comparison, or identity orientation, to provide a more comprehensive understanding of the underlying relationships.

Sixth, data were collected using self-report measures. This method relies on participants' own perceptions, which may be susceptible to influences such as social desirability or recall bias. In addition, common method bias was assessed primarily using Harman's single-factor test. Although this technique is widely applied in behavioral research, it has been criticized for its limited sensitivity in detecting more subtle forms of method variance (Podsakoff et al., 2012). Therefore, the absence of a dominant single factor should not be interpreted as definitive evidence that common method variance is entirely absent.

Given that the data were collected from a single source at a single time point, the possibility of shared method variance cannot be fully ruled out. While procedural remedies such as anonymity assurance and scale separation were implemented during data collection, statistical controls such as marker-variable techniques or CFA-based latent method factor approaches were not employed. This decision was based on the manifest-variable mediation framework adopted in the PROCESS Model 4 analyses, which operates at the observed-variable level rather than the latent level.

Future studies are encouraged to incorporate multi-source data collection designs or advanced statistical controls to further minimize potential method-related bias and strengthen internal validity. In this context, employing objective measurement strategies—such as observational data, performance indicators, or third-party evaluations—may provide a more comprehensive assessment of the constructs and reduce reliance on single-source perceptions.

Seventh, contextual factors such as social media engagement, sponsorship experience, or athletic identity were not included in the model. Such variables can be crucial in forming psychological bonds with a brand. Future studies will explore these factors as mediating or moderating variables, providing a more in-depth understanding of the topic.

In addition to these substantive limitations, the study also presents a methodological constraint related to the measurement procedures. Both the exploratory factor analysis (EFA) and the confirmatory factor analysis (CFA) were conducted on the same sample (N = 338). Although statistical justification was provided for this approach, the absence of cross-sample validation means that the structural stability of the factor solution cannot be independently verified. Conducting EFA and CFA on the same dataset may increase the possibility of capitalization on sample-specific covariance patterns, which could affect the broader structural generalizability of the measurement model.

Moreover, because the sample consisted exclusively of athlete-students and several exclusion criteria were applied, many students in the field of Sports Sciences were not eligible for participation, naturally restricting the achievable sample size. Consequently, the number of participants was insufficient to divide the dataset into independent subsamples for separate EFA and CFA procedures. Accordingly, the structural validity of the measurement model should be interpreted within the contextual boundaries of the present sample.

Despite this limitation, the main structural relationships were examined using latent variable structural equation modeling (SEM), which accounts for measurement error and thus complements the manifest-variable analyses conducted with PROCESS. The inadmissible solution encountered in the second-order latent mediation model—specifically the emergence of a Heywood case—was treated as a statistical estimation constraint rather than as evidence of theoretical inconsistency. To address this issue, the mediation mechanism was evaluated with PROCESS Model 4 using bootstrap confidence intervals, offering a statistically robust assessment of the indirect effect at the observed-variable level. Together, the use of latent SEM and bootstrap-based PROCESS analyses was intended to enhance the robustness of the model estimation within the limits of the available data.

Future research should seek to replicate the factor structure using independent samples and, where possible, employ split-sample or multi-sample validation procedures to strengthen evidence for measurement invariance and structural stability. In addition, longitudinal or multi-sample latent SEM designs may provide a more rigorous test of the proposed mediation model and further clarify the robustness of the structural relationships identified in the present study.

## Data Availability

The raw data supporting the conclusions of this article will be made available by the authors, without undue reservation.
